# Microstructure and Mechanical Property Evolution during Annealing of a Cold-Rolled Metastable Powder Metallurgy High Entropy Alloy

**DOI:** 10.3390/e21090833

**Published:** 2019-08-25

**Authors:** Liangsheng Li, Jingwen Qiu, Wenmin Guo, Bin Liu, Rui Zhou, Zheng Li, Yong Liu

**Affiliations:** 1 State Key Laboratory of Powder Metallurgy, Central South University, Changsha 410083, China; 2 Hunan Provincial Key Laboratory of High Efficiency and Precision Machining of Difficult-to-Cut Material, Hunan University of Science and Technology, Xiangtan 411201, China; 3 YuanMeng Precision Technology (Shenzhen) Institute, Shenzhen 518000, China

**Keywords:** high entropy alloy, powder metallurgy, precipitation, microstructure, mechanical properties

## Abstract

Precipitation strengthening is an effective approach to strengthen high-entropy alloys (HEAs) with a simple face-center-cubic (FCC) structure. In this work, CoCrFeNiMo_0.2_ HEAs were prepared by powder metallurgy, followed by cool rolling and subsequent heat-treatment at different temperatures. The effects of cold working and annealing on microstructure and mechanical properties have been investigated. Results show the fine and dispersed (Cr, Mo)-rich σ phase with a topologically close-packed structure precipitated in the FCC matrix after the prior cold deformation process, which enhanced the mechanical property of the CoCrFeNiMo_0.2_ alloy. The HEA annealed at 600 °C for 48 h had a tensile strength of 1.9 GPa but an elongation which decreased to 8%. The HEA annealed at 800 °C for 12 h exhibited a tensile strength of 1.2 GPa and an elongation of 31%. These outstanding mechanical properties can be attributed to precipitation strengthening and fine-grain strengthening.

## 1. Introduction

Most traditional alloys, such as steels, Al alloys, and nickel-based superalloys, generally consist of one element as their principal component and other additional minor elements to improve their mechanical properties. This conventional limitation on composition design strategy was broken by high-entropy alloys (HEAs), which were proposed by Yeh et al. [1,2]. HEAs are defined as new alloys containing more than five principal elements with each having a concentration between 5 at.% and 35 at.%. Due to their high mixing entropy, HEAs tend to form random solid solutions, such as face-centered cubic (FCC), body-centered cubic (BCC), and even hexagonal close-packed (HCP), resulting in a variety of unusual properties [3,4,5,6,7,8,9,10,11,12,13].

Generally, FCC HEAs exhibit outstanding ductility [3,4,5,6,7,8,9]. However, their yield strength is relatively low. Precipitation strengthening is a good method to strengthen steels and other conventional alloys [14,15,16,17]. It has also been reported that appropriate composition design can promote the formation of secondary precipitation in HEAs through heat treatment, as with other conventional alloys [18,19,20,21,22]. Some literature has indicated that precipitation-strengthening HEAs and steels have achieved both high yield strength and excellent tensile ductility [20,21,22,23]. Different kinds of topologically close-packed (TCP) phases, such as σ phase, μ phase, and Laves phase, etc., have been observed in HEAs [24]. These TCP precipitates have usually increased the mechanical strength of HEAs. However, if precipitates are large in size or unevenly distributed in alloys, they are harmful to the ductility of alloys, resulting in a noticeable degradation of mechanical performance [25,26].

Mo has often been added in HEAs to achieve precipitation strengthening, such as in CoCrCuFeNiMo_x_ [27], HfMo_x_NbTaTiZr [28], and CoCrFeNiMo_x_ [21,29,30,31,32,33,34]. According to the literature [35], the element Mo has limited solubility in CoCrFeNi FCC HEAs. This means that CoCrFeNiMo_x_ HEAs can form intermetallic compounds after heat treatments. At present, precipitated phases such as σ phase or μ phase in CoCrFeNiMo_x_ HEAs have been observed and investigated [29,30,31,32,33,34,35,36]. Such intermetallic compounds have been shown to give FCC HEAs a good combination of high strength and good ductility. Z.P. Lu et al. [21] have reported that the good tensile strength of CoCrFeNiMo_0.3_ HEA is attributed to the fine precipitation of hard intermetallic compounds. In their work, the tensile strength was as high as 1.2 GPa and the ductility was about 19%. In fact, the size, content, and distribution of precipitates have been seen to have great effects on the mechanical performance of CoCrFeNi-based HEAs [20,21,31,32,33,34,35,36,37,38,39,40,41]. It is known that precipitation behavior is closely related to the heat treatment process. To achieve good mechanical properties of CoCrFeNiMo_x_ HEAs, a systematic study of precipitation behavior in different heat treatments is necessary, but research work related to this is still rare.

Since HEAs prepared by powder metallurgy (PM) have not only obtained fine equiaxed crystals but also have avoided component segregation [40,41], powder metallurgy is currently a popular method for preparation of HEAs. It is well known that the cold rolling process can increase deformation energy storage, which can facilitate precipitation in subsequent heat treatments of steel and other alloys [42,43,44,45,46].

In this work, CoCrFeNiMo_0.2_ HEAs are prepared by powder extrusion, followed by cold rolling and subsequent heat-treatment at different temperatures (600–1000 °C). The effects of cold working and annealing on microstructure and mechanical properties are investigated. The effects of precipitation behavior of CoCrFeNiMo_0.2_ HEAs are discussed.

## 2. Materials and Methods

The CoCrFeNiMo_0.2_ HEAs were prepared by the powder metallurgy method in this work. High purity (≥99.9%) metals with a nominal composition of CoCrFeNiMo_0.2_ were melted in a water-cooled copper crucible in an induction-heated vacuum furnace. Then, the melt was dropped through a ceramic tube and atomized by high purity Ar. The atomization pressure was 4 MPa. The metal flow rate and gas flow rate were 50 g/s and 0.25 m^3^/s, respectively. The liquid droplets traveled into the atomization chamber, cooled down, and solidified into spherical pre-alloyed powders. The alloy powders were encapsulated into a stainless steel can with an inner diameter of 60 mm and a length of 150 mm. The encapsulated powder was preheated to 1200 °C for 1 h and subjected to hot extrusion at a velocity of 10 mm/s with an extrusion ratio of 9.5:1 on a hydraulic press. After hot extrusion, the CoCrFeNiMo_0.2_ bars were cooled in the air.

Rectangular samples with a size of 10 mm × 10 mm × 100 mm were cut from the center of the bars by an electric discharge machine. Then, multi-pass rolling with a speed of 30 mm/s was performed at room temperature. The total rolling reduction was about 80% and the reduction per pass was about 8%. The rolled specimens were annealed at 600 °C–1000 °C for different times and subsequently water quenched. The composition of alloy powders was determined by a plasma atomic emission spectrometer (ICAP-7000). The content of oxygen in the CoCrFeNiMo_0.2_ powder was measured by an oxygen analyzer (TCH-600). An optical microscope (OM, Leica DM 4000M) and a field emission scanning electron microscope (FESEM, FEI Nova Nano-230) equipped with an electron backscattered diffraction system (EBSD) were employed to investigate the microstructures of the CoCrFeNiMo_0.2_ alloy. Some of these specimens were characterized by an electron-probe micro-analyzer (EPMA, JEOL JXA-8530F) and a transmission electron microscope (TEM, FEI Tecnai TF-30). The phases of specimens were identified by an X-ray diffractometer (XRD, Bruker D8 ADVANCE). Flat dog-bone tensile specimens with a gauge length of 10 mm, width of 3 mm, and a thickness of 2 mm were cut along the rolling direction. The mechanical properties of all samples were tested using an Instron 3369 materials testing machine at a strain rate of 10^−3^/s.

## 3. Results

### 3.1. Microstructures

**Figure 1:**
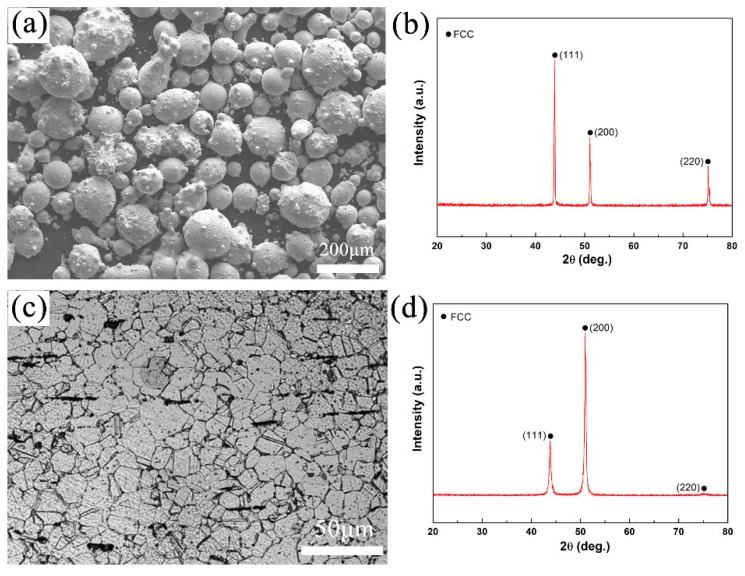
(**a**) SEM image and (**b**) XRD pattern of CoCrFeNiMo_0.2_ powders; (**c**) metallographic photo and (**d**) XRD pattern of the powder metallurgy (PM)-extruded CoCrFeNiMo_0.2_ high-entropy alloy (HEA). Legend: FCC, face-center-cubic.

The chemical composition of the alloy powder obtained by gas atomization is shown in Table 1; the composition is close to the nominal composition of CoCrFeNiMo_0.2_. The oxygen content of the alloy powder is 320 ppm. Figure 1a,b are an SEM photo and XRD pattern of the alloy powder, respectively. It can be seen that the alloy powder is spherical or nearly spherical with a single-phase structure of FCC and a particle size below 150 μm. Figure 1c,d are a metallographic photo and XRD pattern of the CoCrFeNiMo_0.2_ alloy after hot extrusion, respectively. It can be found that the extruded CoCrFeNiMo_0.2_ alloy exhibited an equiaxed grain structure with an average grain size of about 20 μm. After powder hot extrusion, the CoCrFeNiMo_0.2_ alloy still remained an FCC phase. 

**Figure 2:**
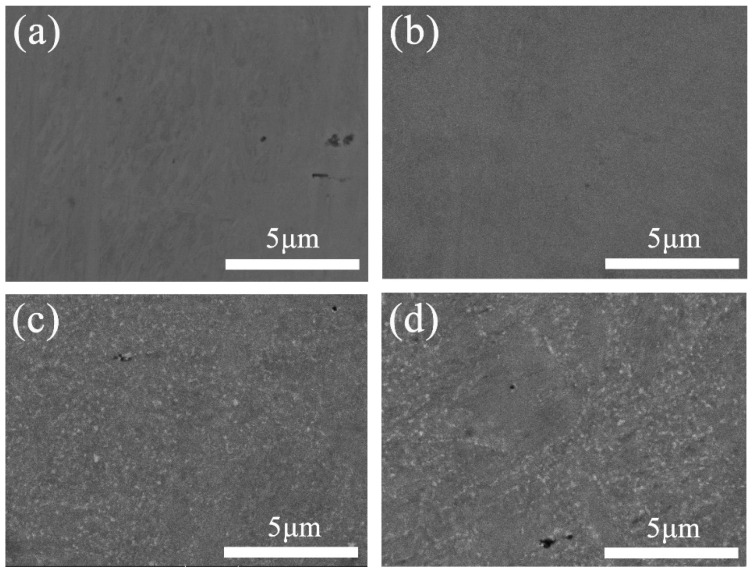
SEM images of the powder metallurgical CoCrFeNiMo_0.2_ alloy under various conditions: (**a**) cold-rolled; annealed at (**b**) 600 °C for 12 h, (**c**) 600 °C for 24 h, and (**d**) 600 °C for 48 h.

Figure 2 shows the microstructures of the CoCrFeNiMo_0.2_ HEA under different conditions. Figure 2a indicates that the cold-rolled powder metallurgical CoCrFeNiMo_0.2_ HEA was compact and uniform. Figure 2b–d show backscattered electron (BSE) images of the CoCrFeNiMo_0.2_ HEA annealed at 600 °C under different holding times. No precipitate was found in the HEA shown in Figure 2b. The precipitate was observed in the HEA when the holding time reached 24 h, as shown in Figure 2c. When the holding time was extended to 48 h, the volume fraction of the precipitate increased but the size of the particles did not increase significantly, as shown in Figure 2d.

**Figure 3:**
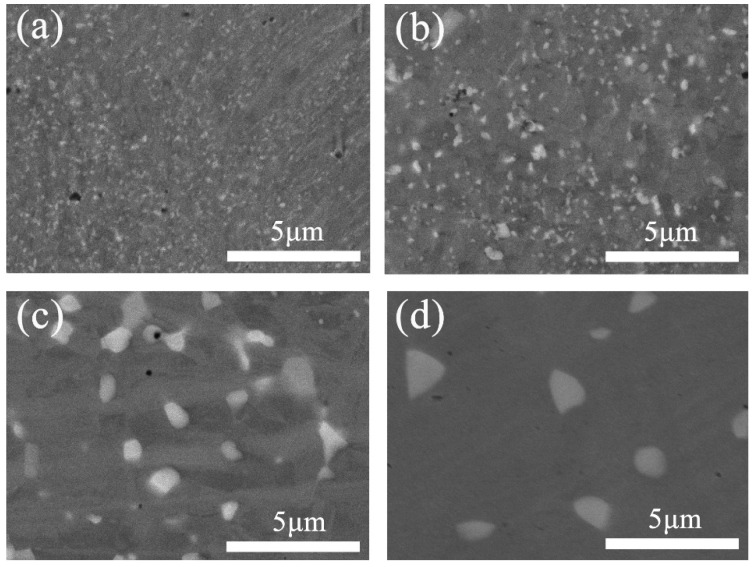
SEM images of the cold-rolled CoCrFeNiMo_0.2_ alloy annealed under various conditions: (**a**) 700 °C for 12 h, (**b**) 800 °C for 12 h, (**c**) 900 °C for 12 h, and (**d**) 1000 °C for 12 h.

Figure 3 shows SEM images of the cold-rolled CoCrFeNiMo_0.2_ HEAs annealed for 12 h at different temperatures. The precipitate was observed in the HEA under the annealing temperature of 700 °C, as in Figure 3a. The size of the precipitate grew as the temperature increased from 700 °C to 1000 °C.

**Figure 4:**
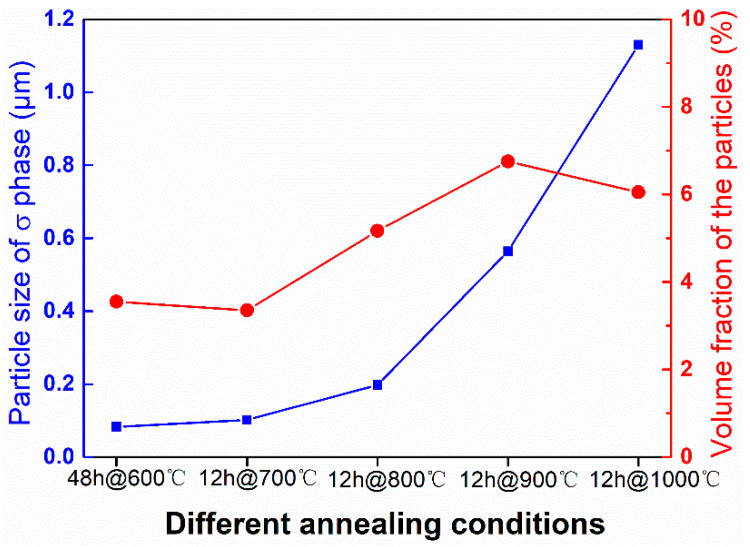
Variation in the average size and volume fraction of σ precipitate with different annealing conditions.

Figure 4 shows the statistical results of the variation in the average size and volume fraction of the σ precipitate under different annealing conditions. The statistical results of the alloy annealed at 600 °C for 48 h are close to those of the alloy annealed at 700 °C for 12 h. The average size of the σ precipitate is less than 0.2 μm when annealed at 700 °C for 12 h. As the annealing temperature increases, the average size of the σ precipitates increases. When the annealing temperature reaches 1000 °C, the average size of the σ precipitate exceeds 1 μm. The volume fraction of the σ precipitate increases first and then decreases as the annealing temperature increases, and the maximum does not exceed 8%. Therefore, a uniformly dispersed σ nano-precipitate is obtained in the CoCrFeNiMo_0.2_ HEA via cold-rolled and annealing processes.

**Figure 5:**
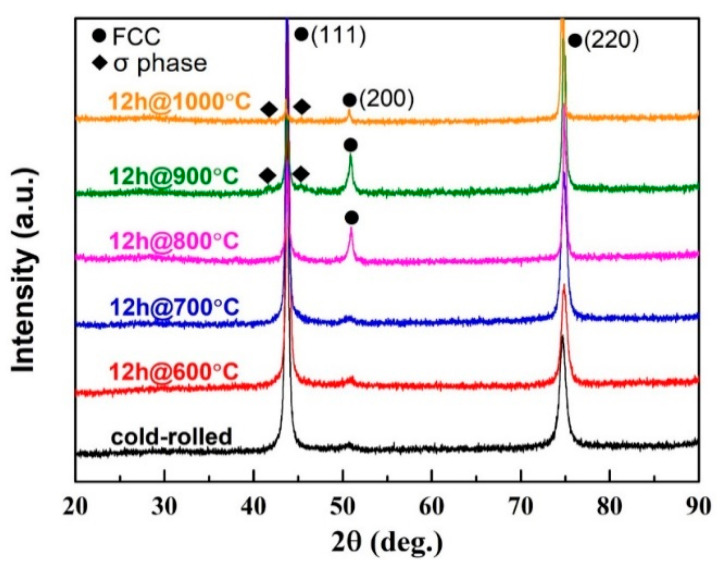
X-ray diffraction patterns of the cold-rolled powder metallurgical CoCrFeNiMo_0.2_ alloy under various conditions.

X-ray diffraction patterns of the cold-rolled and annealed CoCrFeNiMo_0.2_ HEAs are shown in Figure 5. Diffraction peaks from the matrix with a single FCC phase are observed in all the samples. When the annealing temperature reaches 900 °C, low-intensity diffraction peaks from the σ phase can be detected besides the FCC phase of the matrix. Though a number of precipitates can be found in Figure 3, the intensity diffraction peak of the σ phase is low. A similar phenomenon of low-intensity diffraction peaks of the σ phase has been discussed in other paper [30]. The main reason for this phenomenon may be that the content of the σ phase precipitate is too low. The small size of the σ phase precipitate in this work may be another reason.

**Figure 6:**
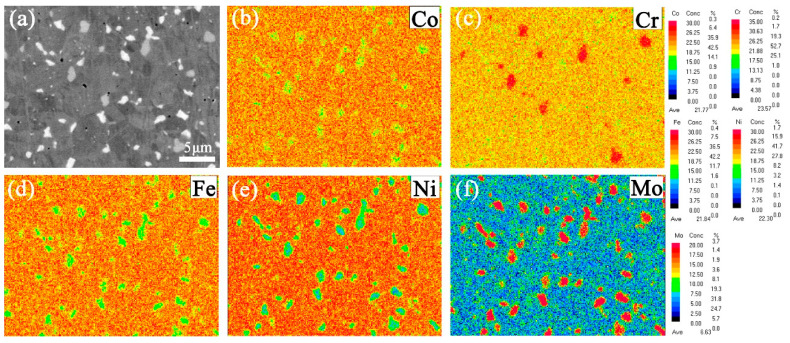
Electron-probe micro-analyzer (EPMA)-backscattered electron (BSE) image (**a**) and elemental maps for Co (**b**), Cr (**c**), Fe (**d**), Ni (**e**), and Mo (**f**) of the cold-rolled powder metallurgical CoCrFeNiMo_0.2_ alloy specimen annealed at 900 °C for 12 h.

Figure 6 shows a BSE image and EPMA elemental maps of the specimen annealed at 900 °C for 12 h. As shown in Figure 6c,f, it is clear that the Cr and Mo elements are enriched in the precipitate. On the contrary, the Co, Fe, and Ni elements are enriched in the matrix. In combination with the BSE image shown in Figure 6a, there seem to be two kinds of precipitates with different contrasts in the alloy. The chemical composition of the precipitates which show different colors in Figure 6a, which was measured by EPMA, are given in Table 2. The results show that the composition of the matrix is consistent with the nominal composition and that the composition of the white precipitate is indeed different from that of the gray precipitate. However, the sum content of the Cr and Mo elements in the white precipitate is almost the same as that in the gray precipitate. The total of the Cr and Mo elements are 50% and the rest of the elements (Fe, Cr, and Ni) are 50%.

**Figure 7:**
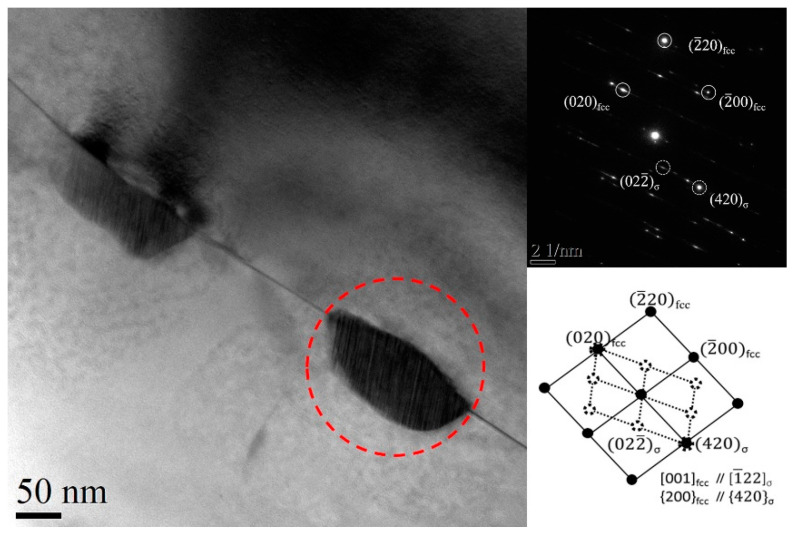
Transmission electron microscopy (TEM) image and selected area electron diffraction (SAED) pattern of the cold-rolled powder metallurgical CoCrFeNiMo_0.2_ alloy specimen annealed at 800 °C for 12 h.

Figure 7 shows a TEM image of the specimen annealed at 800 °C for 12 h. It can be seen that the size of the precipitate particles is about 100 nm. The selected area electron diffraction (SAED) pattern and calibrated result are also shown in Figure 7. The main diffraction spots reveal that the matrix is an FCC phase and the additional diffraction spots observed confirm that the precipitate is the σ phase with a topological close-packed (TCP) structure [21].

**Figure 8:**
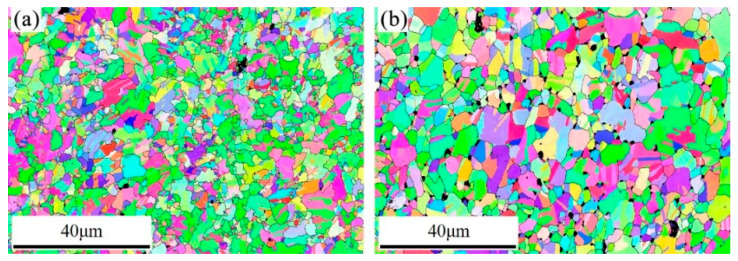
IPF map of the cold-rolled powder metallurgical CoCrFeNiMo_0.2_ alloy specimen annealed at (**a**) 800 °C for 12 h and (**b**) 900 °C for 12 h.

Figure 8 shows an inverse pole figure (IPF) map of the CoCrFeNiMo_0.2_ HEA annealed at different temperatures. It is obvious that the annealed alloy exhibits an equiaxed grain structure. The average grain size of the alloy annealed at 800 °C is about 5.9 μm. When the annealing temperature reaches 900 °C, the average grain size is about 9.6 μm. This indicates that the grain size of the alloy does not increase significantly after annealing.

### 3.2. Mechanical Properties

**Figure 9:**
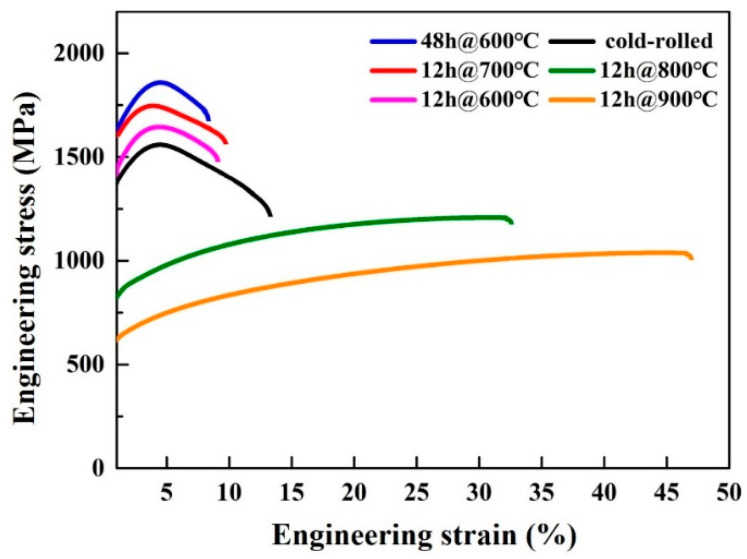
Room-temperature tensile engineering strain-stress curves of the cold-rolled powder metallurgical CoCrFeNiMo_0.2_ alloy under various conditions.

Figure 9 presents engineering stress-strain curves of the cold-rolled and annealed CoCrFeNiMo_0.2_ HEAs at different temperatures. The yield strength (YS), ultimate tensile strength (UTS), and elongation-to-failure (EL) are summarized in Table 3. The YS and UTS of the HEA annealed at 600 °C for 12 h are 1624 MPa and 1779 MPa, respectively, while the EL is about 9.5%. When the annealing time increases from 12 h to 48 h, the UTS of the HEA increases from 1779 MPa to 1869 MPa but the EL drops to 8%. The YS and UTS of the HEAs gradually decrease and the EL of the HEAs increases as the annealing temperature increases. The EL of the HEA annealed at 900 °C for 12 h reaches up to 46%. It is worth noting that some curves show that the alloy undergoes a prolonged strain softening stage after a short hardening stage. This phenomenon may be related to the early occurrence of necking instability in these alloys during the tensile test [3].

**Figure 10:**
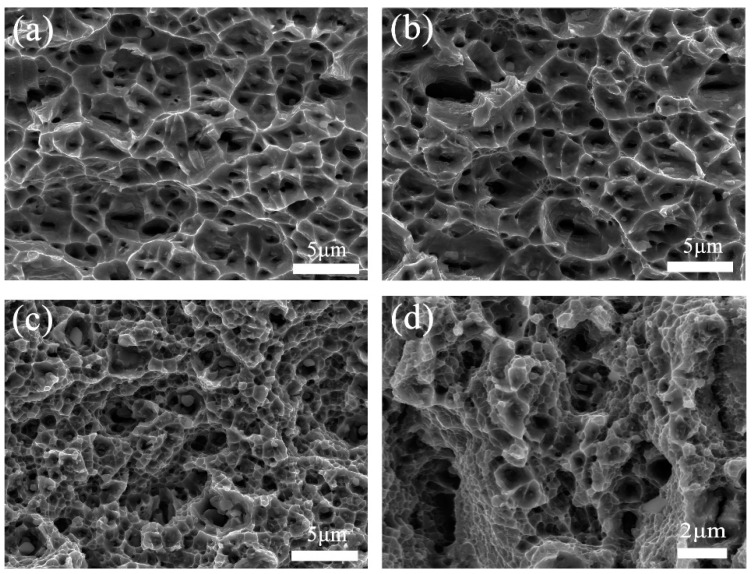
The fracture surfaces of the cold-rolled powder metallurgical CoCrFeNiMo_0.2_ alloy under various conditions: (**a**) cold-rolled; annealed at (**b**) 600 °C for 12 h, (**c**) 600 °C for 48 h, and (**d**) 800 °C for 12 h.

Figure 10 shows the fracture morphologies of the cold-rolled and annealed CoCrFeNiMo_0.2_ HEAs under various conditions. Many “small facets” with different orientation can be observed in Figure 10a,b. The fracture mode of CoCrFeNiMo_0.2_ belongs to the intergranular fracture. A large number of dimple joints can be observed in Figure 10c,d. This indicates that the fracture mode changes from intergranular fracture to a transgranular-toughness fracture with increase in annealing temperature or holding time.

## 4. Discussion

Shun et al. [30] first reported that the (Cr, Mo)-rich σ phase existed in the CoCrFeNiMo_0.5_ HEA. Many studies [31,32,33,34,35,36] have indicated the composition of the σ phase corresponds to stoichiometric (Cr, Mo)_5_(Co, Fe, Ni)_5_. The chemical compositions of the gray precipitate and the white precipitate, as given in Table 2, are both consistent with the stoichiometric ratio mentioned above. Based on the SAED pattern of Figure 7, it can be concluded that the precipitate in the present CoCrFeNiMo_0.2_ HEA is the σ phase with a TCP structure. A recent study [35] about the thermodynamic calculation of CoCrFeNiMo_x_ HEAs pointed out that the previous isopleth phase diagram of (CoCrFeNi)-Mo is unreasonable and Cr should be analyzed separately, just like the Mo element, when drawing phase diagrams. The (CoFeNi)-Mo-Cr isothermal phase diagram should be more reasonable. According to the isothermal phase diagram, the precipitation phase in the CoCrFeNiMo_0.2_ HEA can only be the σ phase under any temperature. It is well known that backscattered electrons display intensity or contrast variations of the microstructure which depend on the atomic number (Z) of the element. Based on the results of Figure 6 and Table 2, the precipitate phase with more Mo and Cr will exhibit bright contrast. The difference in color of the precipitates in this work can be attributed to the difference in chemical composition. Therefore, the precipitates of two different colors in the annealed CoCrFeNiMo_0.2_ HEA are supposed to be the same crystal type of precipitate.

In this work, the UST of the CoCrFeNiMo_0.2_ HEA annealed at 600 °C for 48 h is as high as 1.9 GPa, which has never been reported before. As the annealing temperature increases, the CoCrFeNiMo_0.2_ HEA annealed at 900 °C for 12 h shows an excellent ductility of 46% EL, but the UST decreases to 1039 MPa. The tensile properties of the present CoCrFeNiMo_0.2_ HEA annealed at different temperatures obey typical strength and ductility trade-off behavior. The EL of the CoCrFeNiMo_0.2_ HEA annealed at 800 °C for 12 h is 31%, and the UST still maintains 1.2 GPa. Compared with other heat treatment conditions, this is the best comprehensive mechanical performance of CoCrFeNiMo_0.2_ HEA. Table 4 gives the room temperature tensile properties of CoCrFeNi-based HEAs reported recently. It can be found that the comprehensive mechanical properties of the CoCrFeNiMo_0.2_ HEA after cold-rolling (80%) and annealing (800 °C/12 h) in this work are still attractive. In particular, compared with previous precipitation-strengthened CoCrFeNi-based HEAs [20,21], CoCrFeNiMo_0.2_ HEA annealed at 800 °C for 12 h achieves a higher tensile strength at a lower annealing temperature, or superior ductility at the same tensile strength. Moreover, the CoCrFeNiMo_0.2_ HEA with a lower Mo content in this work may also help to develop more cost-effective high strength HEAs in the application field. 

The formation of hard second phases can effectively strengthen alloys; this has been reported for many different alloys [17,18,19,20,21,22,23,24]. Combined with the statistical results of Figure 4, it can be concluded that size and content of the precipitates are closely related to the mechanical properties of the alloy. The nano-precipitates can effectively improve the mechanical properties of the alloy. It has been reported that precipitation of second phase particles can pin grain boundaries, suppress grain growth, and result in the formation of fine-grain microstructures in the FCC type HEAs [37,38,39]. According to Figure 1c, the average grain size of the extruded alloy is about 20 μm. The grain size of the alloy after cold-rolling and annealing at 900 °C is 9.6 μm based on the EBSD result shown in Figure 8b. Though the alloy is annealed above the recrystallization temperature, the grain size of the alloy is not significantly increased, which can be attributed to the pinning effect of the precipitates. Therefore, according to the Hall-Petch mechanism, fine-grain strengthening should be another positive factor of the outstanding mechanical properties of the CoCrFeNiMo_0.2_ HEA. 

On the other hand, as shown in Figure 1c, PM extruded HEA with fine and equiaxed grains, providing a basis for cold-rolling (80% deformation). It is known that the large cold deformation would break the grain and bring in enough defects, such as subgrain-boundaries and dislocations in the microstructure. These defects can work as precipitation sites and promote the precipitation of the σ phase. Then, the finer and more uniform σ precipitates may enhance the mechanical properties of CoCrFeNiMo_0.2_ HEA further. This means that the PM extrusion preparation method combined with cold-rolling and subsequent annealing could be regarded as a promising means of fabricating high performance HEAs.

## 5. Conclusions

(1) The powder metallurgy extruded CoCrFeNiMo_0.2_ HEA has a single FCC structure. After cold-rolling and heat-treatment, a (Cr, Mo)-rich σ with TCP structure can precipitate in the HEA.
(2) CoCrFeNiMo_0.2_ HEA annealed at 800 °C for 12 h achieved the best overall mechanical property of 1.2 GPa tensile strength and 31% fracture strain, which can be expected to be a good candidate of high-strength and high-tenacity structural materials.
(3) The excellent mechanical properties may be attributed to precipitation strengthening and fine grain strengthening. The present process could be considered as a promising means of fabricating high performance HEAs.

## Figures and Tables

**Table 1:** Chemical composition of the CoCrFeNiMo_0.2_ powders.

Element (at.%)	Co	Cr	Fe	Ni	Mo
Powders	22.33	23.96	25.05	23.12	5.54

**Table 2:** Chemical composition of phases in the CoCrFeNiMo_0.2_ HEA annealed at 900 °C for 12 h shown in Figure 6a (at.%, measured by EPMA).

Phase	Co	Cr	Fe	Ni	Mo
FCC (matrix)	23.71	23.98	23.51	24.44	4.36
White precipitate	21.08	25.73	17.61	11.38	24.20
Gray precipitate	19.55	35.78	17.51	11.44	15.72

**Table 3:** The yield strength (YS), ultimate tensile strength (UTS), and elongation-to-failure (EL) for the powder metallurgical CoCrFeNiMo_0.2_ alloy annealed under different conditions.

Samples	YS (MPa)	UTS (MPa)	EL (%)
Cold-rolled	1392 ± 20	1589 ± 9	13.5 ± 1.5
600 °C (12 h)	1624 ± 10	1779 ± 5	9.5 ± 0.5
600 °C (48 h)	1631 ± 10	1869 ± 8	8 ± 1
700 °C (12 h)	1448 ± 12	1645 ± 11	10 ± 0.8
800 °C (12 h)	834 ± 10	1208 ± 25	31 ± 1.6
900 °C (12 h)	622 ± 8	1039 ± 3	46 ± 1.5

**Table 4:** The post-treatment UTS and EL values for CoCrFeNi-based HEAs which have been reported.

HEAs	Post-Treatment	UTS (MPa)	EL (%)
CoCrFeMnNi [3]	Cold-rolled and recrystallized	600	60
CoCrFeNiNb_0.4_ [6]	No treatment	1004	1.3
(FeCoNiCr)_94_Ti_2_Al_4_ [20]	Cold-rolled (30%) and annealed (800 °C/18 h)	1098	39
CoCrFeNiMo_0.3_ [21]	Cold-rolled (60%) and annealed (850 °C/1 h)	1187	18.9
CoCrFeNiMo_0.15_ [33]	Torsional-treated 360°	1000	10
Current work	Cold-rolled (80%) and annealed (800 °C/12 h)	1208	31

## References

[1] Huang P.K., Yeh J.W., Shun T.T., Chen S.K. (2004). Multi-Principal-Element Alloys with Improved Oxidation and Wear Resistance for Thermal Spray Coating. Adv. Eng. Mater..

[2] Yeh J.W., Chen S.K., Lin S.J., Gan J.Y., Chin T.S., Shun T.T., Tsau C.H., Chang S.Y. (2004). Nanostructured high-entropy alloys with multiple principal elements: novel alloy design concepts and outcomes. Adv. Eng. Mater..

[3] Otto F., Dlouhý A., Somsen C., Bei H., Eggeler G., George E.P. (2013). The influences of temperature and microstructure on the tensile properties of a CoCrFeMnNi high-entropy alloy. Acta Mater..

[4] Gludovatz B., Hohenwarter A., Catoor D., Chang E.H., George E.P., Ritchie R.O. (2014). A fracture-resistant high-entropy alloy for cryogenic applications. Science.

[5] Youssef K.M., Zaddach A.J., Niu C., Irving D.L., Koch C.C. (2015). A Novel Low-Density, High-Hardness, High-entropy Alloy with Close-packed Single-phase Nanocrystalline Structures. Mater. Res. Lett..

[6] Wu Q., Wang Z., Zheng T., Chen D., Yang Z., Li J., Kai J., Wang J. (2019). A casting eutectic high entropy alloy with superior strength-ductility combination. Mater. Lett..

[7] Yu Y., He F., Qiao Z., Wang Z., Liu W., Yang J. (2019). Effects of temperature and microstructure on the triblogical properties of CoCrFeNiNbx eutectic high entropy alloys. J. Alloys Compd..

[8] He J.Y., Liu W.H., Wang H., Wu Y., Liu X.J., Nieh T.G., Lu Z.P. (2014). Effects of Al addition on structural evolution and tensile properties of the FeCoNiCrMn high-entropy alloy system. Acta Mater..

[9] Zhang Y., Zuo T.T., Tang Z., Gao M.C., Dahmen K.A., Liaw P.K., Lu Z.P. (2014). Microstructures and properties of high-entropy alloys. Prog. Mater. Sci..

[10] Zou Y., Ma H., Spolenak R. (2015). Ultrastrong ductile and stable high-entropy alloys at small scales. Nat. Commun..

[11] Senkov O.N., Senkova S.V., Woodward C. (2014). Effect of aluminum on the microstructure and properties of two refractory high-entropy alloys. Acta Mater..

[12] Senkov O.N., Scott J.M., Senkova S.V., Miracle D.B., Woodward C.F. (2011). Microstructure and room temperature properties of a high-entropy TaNbHfZrTi alloy. J. Alloys Compd..

[13] Takeuchi A., Amiya K., Wada T., Yubuta K., Wei Z. (2014). High-entropy alloys with a hexagonal close-packed structure designed by equi-atomic alloy strategy and binary phase diagrams. JOM.

[14] Jiang S., Wang H., Wu Y., Liu X., Chen H., Yao M., Gault B., Ponge D., Raabe D., Hirata A. (2017). Ultrastrong steel via minimal lattice misfit and high-density nanoprecipitation. Nature.

[15] Sun Z., Liebscher C.H., Huang S., Teng Z., Song G., Wang G., Asta M., Rawlings M., Fine M.E., Liaw P.K. (2013). New design aspects of creep-resistant NiAl-strengthened ferritic alloys. Scr. Mater..

[16] Teng Z.K., Ghosh G., Miller M.K., Huang S., Clausen B., Brown D.W., Liaw P.K. (2012). Neutron-diffraction study and modeling of the lattice parameters of a NiAl-precipitate-strengthened Fe-based alloy. Acta Mater..

[17] Kim S.-H., Kim H., Kim N.J. (2015). Brittle intermetallic compound makes ultrastrong low-density steel with large ductility. Nature.

[18] Pickering E.J., Munoz-Moreno R., Stone H.J., Jones N.G. (2016). Precipitation in the equiatomic high-entropy alloy CrMnFeCoNi. Scr. Mater..

[19] He F., Wang Z., Niu S., Wu Q., Li J., Wang J., Liu C.T., Dang Y. (2016). Strengthening the CoCrFeNiNb0.25 high entropy alloy by FCC precipitate. J. Alloys Compd..

[20] He J.Y., Wang H., Huang H.L., Xu X.D., Chen M.W., Wu Y., Liu X.J., Nieh T.G., An K., Lu Z.P. (2016). A precipitation-hardened high-entropy alloy with outstanding tensile properties. Acta Mater..

[21] Liu W.H., Lu Z.P., He J.Y., Luan J.H., Wang Z.J., Liu B., Liu Y., Chen M.W., Liu C.T. (2016). Ductile CoCrFeNiMox high entropy alloys strengthened by hard intermetallic phases. Acta Mater..

[22] Li Z., Pradeep K.G., Deng Y., Raabe D., Tasan C.C. (2016). Metastable high-entropy dual-phase alloys overcome the strength–ductility trade-off. Nature.

[23] Liu P.P., Zhao M.Z., Zhu Y.M., Bai J.W., Wan F.R., Zhan Q. (2013). Effects of carbide precipitate on the mechanical properties and irradiation behavior of the low activation martensitic steel. J. Alloys Compd..

[24] Otto F., Yang Y., Bei H., George E.P. (2013). Relative effects of enthalpy and entropy on the phase stability of equiatomic high-entropy alloys. Acta Mater..

[25] Rehman H.u., Durst K., Neumeier S., Parsa A.B., Kostka A., Eggeler G., Göken M. (2015). Nanoindentation studies of the mechanical properties of the μ phase in a creep deformed Re containing nickel-based superalloy. Mater. Sci. Eng. A.

[26] Rae C.M.F., Reed R.C. (2001). The precipitation of topologically close-packed phases in rhenium-containing superalloys. Acta Mater..

[27] Yang Q., Tang Y., Wen Y., Zhang Q., Deng D., Nai X. (2018). Microstructures and properties of CoCrCuFeNiMox high-entropy alloys fabricated by mechanical alloying and spark plasma sintering. Powder Metall..

[28] Juan C.-C., Tseng K.-K., Hsu W.-L., Tsai M.-H., Tsai C.-W., Lin C.-M., Chen S.-K., Lin S.-J., Yeh J.-W. (2016). Solution strengthening of ductile refractory HfMoxNbTaTiZr high-entropy alloys. Mater. Lett..

[29] Shun T.-T., Hung C.-H., Lee C.-F. (2010). Formation of ordered/disordered nanoparticles in FCC high entropy alloys. J. Alloys Compd..

[30] Shun T.-T., Chang L.-Y., Shiu M.-H. (2012). Microstructure and mechanical properties of multiprincipal component CoCrFeNiMox alloys. Mater. Charact..

[31] Shun T.-T., Chang L.-Y., Shiu M.-H. (2013). Age-hardening of the CoCrFeNiMo0.85 high-entropy alloy. Mater. Charact..

[32] Csaki I., Karlsdottir S.N., Serghiuta S., Popescu G., Buzatu M., Geambazu L.E., Manea C.A. (2017). CoCrFeNiMo high entropy alloy produced by solid state processing. Key Eng. Mater..

[33] Wu W., Guo L., Liu B., Ni S., Liu Y., Song M. (2017). Effects of torsional deformation on the microstructures and mechanical properties of a CoCrFeNiMo0.15 high-entropy alloy. Philos. Mag..

[34] Bae J.W., Park J.M., Moon J., Choi W.M., Lee B.-J., Kim H.S. (2019). Effect of μ-precipitates on the microstructure and mechanical properties of non-equiatomic CoCrFeNiMo medium-entropy alloys. J. Alloys Compd..

[35] Wu Q., Wang Z., He F., Li J., Wang J. (2018). Revealing the Selection of σ and μ Phases in CoCrFeNiMox High Entropy Alloys by CALPHAD. J. Phase Equilib. Diff..

[36] Cai B., Liu B., Kabra S., Wang Y., Yan K., Lee P.D., Liu Y. (2017). Deformation mechanisms of Mo alloyed FeCoCrNi high entropy alloy: In situ neutron diffraction. Acta Mater..

[37] Yasuda H.Y., Miyamoto H., Cho K., Nagase T. (2017). Formation of ultrafine-grained microstructure in Al0.3 CoCrFeNi high entropy alloys with grain boundary precipitates. Mater. Lett..

[38] Stepanov N.D., Shaysultanov D.G., Chernichenko R.S., Ikornikov D.M., Sanin V.N., Zherebtsov S.V. (2018). Mechanical properties of a new high entropy alloy with a duplex ultra-fine grained structure. Mater. Sci. Eng. A.

[39] Klimova M.V., Shaysultanov D.G., Zherebtsov S.V., Stepanov N.D. (2019). Effect of second phase particles on mechanical properties and grain growth in a CoCrFeMnNi high entropy alloy. Mater. Sci. Eng. A.

[40] Liu B., Wang J., Liu Y., Fang Q., Wu Y., Chen S., Liu C.T. (2016). Microstructure and mechanical properties of equimolar FeCoCrNi high entropy alloy prepared via powder extrusion. Intermetallics.

[41] Liu B., Xu L., Liu Y., Wang J., Wang J., Fang Q. (2018). Effect of cold working and annealing on microstructure and properties of powder metallurgy high entropy alloy. Sci. China Technol. Sci..

[42] Ghosh P., Ray R.K., Bhattacharya B., Bhargava S. (2006). Precipitation and texture formation in two cold rolled and batch annealed interstitial-free high strength steels. Scripta Mater..

[43] Heo N.J., Nagasaka T., Muroga T. (2004). Recrystallization and precipitation behavior of low-activation V–Cr–Ti alloys after cold rolling. J. Nucl. Mater..

[44] Hou J., Zhang M., Ma S., Liaw P.K., Zhang Y., Qiao J. (2017). Strengthening in Al0.25CoCrFeNi high-entropy alloys by cold rolling. Mater. Sci. Eng. A.

[45] Hou J., Shi X., Qiao J., Zhang Y., Liaw P.K., Wu Y. (2019). Ultrafine-grained dual phase Al0.45CoCrFeNi high-entropy alloys. Mater. Des..

[46] Mei Y., Liu Y., Liu C., Li C., Yu L., Guo Q., Li H. (2015). Effects of cold rolling on the precipitation kinetics and the morphology evolution of intermediate phases in Inconel 718 alloy. J. Alloys Compd..

